# *N*′-Cyclo­hexyl­idene-2-hydroxy­benzo­hydrazide

**DOI:** 10.1107/S1600536809007636

**Published:** 2009-03-06

**Authors:** Deyun Liu

**Affiliations:** a Liaocheng Vocational and Technical College, Liaocheng, 252059, People’s Republic of China

## Abstract

In the title mol­ecule, C_13_H_16_N_2_O_2_, the cyclo­hexyl­idene ring adopts a chair conformation. The intra­molecular N—H⋯O hydrogen bond influences the mol­ecular conformation: the benzene ring and the mean plane of the central C(O)NHN fragment form a dihedral angle of 4.9 (1) Å. In the crystal, inter­molecular O—H⋯O hydrogen bonds link the mol­ecules into chains propagated along [001].

## Related literature

For properties of Shiff-base derivatives, see Sreeja *et al.* (2003 ▶). For a related structure, see Luo *et al.* (2007 ▶).
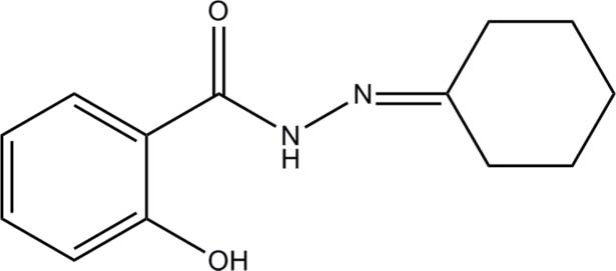


## Experimental

### 

#### Crystal data

C_13_H_16_N_2_O_2_
*M* *_r_* = 232.28
Monoclinic, 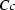

*a* = 18.376 (2) Å
*b* = 5.3386 (10) Å
*c* = 12.9435 (15) Å
β = 102.241 (2)°
*V* = 1240.9 (3) Å^3^
*Z* = 4
Mo *K*α radiation
μ = 0.09 mm^−1^
*T* = 293 K
0.39 × 0.29 × 0.27 mm

#### Data collection

Bruker SMART APEX CCD area-detector diffractometer
Absorption correction: multi-scan (*SADABS*; Sheldrick, 1996 ▶) *T*_min_ = 0.968, *T*_max_ = 0.977
2969 measured reflections
1090 independent reflections
898 reflections with *I* > 2σ(*I*)
*R*_int_ = 0.024

#### Refinement

*R*[*F* ^2^ > 2σ(*F* ^2^)] = 0.047
*wR*(*F* ^2^) = 0.138
*S* = 1.08
1090 reflections
154 parameters
2 restraints
H-atom parameters constrained
Δρ_max_ = 0.28 e Å^−3^
Δρ_min_ = −0.15 e Å^−3^

### 

Data collection: *SMART* (Siemens, 1996 ▶); cell refinement: *SAINT* (Siemens, 1996 ▶); data reduction: *SAINT*; program(s) used to solve structure: *SHELXS97* (Sheldrick, 2008 ▶); program(s) used to refine structure: *SHELXL97* (Sheldrick, 2008 ▶); molecular graphics: *SHELXTL* (Sheldrick, 2008 ▶); software used to prepare material for publication: *SHELXTL*.

## Supplementary Material

Crystal structure: contains datablocks I, global. DOI: 10.1107/S1600536809007636/cv2521sup1.cif

Structure factors: contains datablocks I. DOI: 10.1107/S1600536809007636/cv2521Isup2.hkl

Additional supplementary materials:  crystallographic information; 3D view; checkCIF report

## Figures and Tables

**Table 1:** Hydrogen-bond geometry (Å, °)

*D*—H⋯*A*	*D*—H	H⋯*A*	*D*⋯*A*	*D*—H⋯*A*
N1—H1⋯O2	0.86	1.97	2.655 (4)	136
O2—H2⋯O1^i^	0.82	2.15	2.704 (4)	125

Symmetry code: (i) 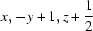.

**Fig. 1.**
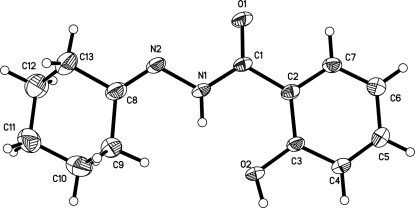
The molecular structure of (I) with the atomic numbering scheme and 30% probability displacement ellipsoids.

## supplementary crystallographic information

### Comment

Chemistry of Schiff bases has been intensively investigated in recent years, owing to their coordination properties and diverse applications. Schiff base derivatives and their complexes have been studied for their antifungal and antibacterial activ- ity, and as antiviral drugs (Sreeja *et al.*, 2003). In this paper, we present the crystal structure of the title compound, (I), which was synthesized by the reaction of cyclohexanone and salicyloyl hydrazide.

In (I) (Fig. 1), the bond lengths and angles are normal and comparable to those observed in the compound reported by Luo *et al.*  (2007). The cyclohexylidene ring adopts a chair conformation. Intramolecular N—H···O hydrogen bond (Table 1) influences the molecular conformationthe - dihedral angle between the benzene ring and the plane C1/N1/N2 is 4.9 (1) Å. The plane C1/N1/N2 and ring C8-C13 form a dihedral angle of 37.7 (3) Å. Intermolecular O—H···O hydrogen bonds (Table 1) link the molecules into chains propagated in direction [001].

### Experimental

Salicyloyl hydrazide (5 mmol) and cyclohexanone (5 mmol),20 ml e nthanol were mixed in 50 ml flash. After stirring 30 min at 353 K, the mixture then cooling slowly to room temperature and affording the title compound, then recrystallized from ethanol, affording the title compound as a red crystalline solid. Elemental analysis: calculated for C_13_H_16_N_2_O_2_: C 67.22, H 6.94, N 12.06%; found: C 67.29, H 6.85, N 12.24%.

### Refinement

All H atoms were placed in geometrically idealized positions (N—H 0.86 Å, O—H 0.82 Å and C—H=0.93–0.97 Å) and treated as riding on their parent atoms, with *U*_iso_(H) = 1.2-1.5*U*_eq_ of the parent atom. In the absence of any significant anomalous scatterers in the molecule, 330 Friedel pairs were merged before the final refinement.

### Crystal data

**Table:**

C_13_H_16_N_2_O_2_	*F*(000) = 496
*M**_r_* = 232.28	*D*_x_ = 1.243 Mg m^−^^3^
Monoclinic, *C**c*	Mo *K*α radiation, λ = 0.71073 Å
*a* = 18.376 (2) Å	Cell parameters from 1322 reflections
*b* = 5.3386 (10) Å	θ = 3.2–27.5°
*c* = 12.9435 (15) Å	µ = 0.09 mm^−^^1^
β = 102.241 (2)°	*T* = 293 K
*V* = 1240.9 (3) Å^3^	Block, red
*Z* = 4	0.39 × 0.29 × 0.27 mm

### Data collection

**Table:**

Bruker SMART Apex CCD area-detector diffractometer	1090 independent reflections
Radiation source: fine-focus sealed tube	898 reflections with *I* > 2σ(*I*)
graphite	*R*_int_ = 0.024
phi and ω scans	θ_max_ = 25.0°, θ_min_ = 2.3°
Absorption correction: multi-scan (*SADABS*; Sheldrick, 1996)	*h* = −21→11
*T*_min_ = 0.968, *T*_max_ = 0.977	*k* = −6→6
2969 measured reflections	*l* = −15→15

### Refinement

**Table:**

Refinement on *F*^2^	Primary atom site location: structure-invariant direct methods
Least-squares matrix: full	Secondary atom site location: difference Fourier map
*R*[*F*^2^ > 2σ(*F*^2^)] = 0.047	Hydrogen site location: inferred from neighbouring sites
*wR*(*F*^2^) = 0.138	H-atom parameters constrained
*S* = 1.08	*w* = 1/[σ^2^(*F*_o_^2^) + (0.0866*P*)^2^ + 0.3353*P*] where *P* = (*F*_o_^2^ + 2*F*_c_^2^)/3
1090 reflections	(Δ/σ)_max_ < 0.001
154 parameters	Δρ_max_ = 0.28 e Å^−^^3^
2 restraints	Δρ_min_ = −0.15 e Å^−^^3^

### Special details

**Table:**

Geometry. All e.s.d.'s (except the e.s.d. in the dihedral angle between two l.s. planes) are estimated using the full covariance matrix. The cell e.s.d.'s are taken into account individually in the estimation of e.s.d.'s in distances, angles and torsion angles; correlations between e.s.d.'s in cell parameters are only used when they are defined by crystal symmetry. An approximate (isotropic) treatment of cell e.s.d.'s is used for estimating e.s.d.'s involving l.s. planes.
Refinement. Refinement of *F*^2^ against ALL reflections. The weighted *R*-factor *wR* and goodness of fit *S* are based on *F*^2^, conventional *R*-factors *R* are based on *F*, with *F* set to zero for negative *F*^2^. The threshold expression of *F*^2^ > σ(*F*^2^) is used only for calculating *R*-factors(gt) *etc*. and is not relevant to the choice of reflections for refinement. *R*-factors based on *F*^2^ are statistically about twice as large as those based on *F*, and *R*- factors based on ALL data will be even larger.

### Fractional atomic coordinates and isotropic or equivalent isotropic displacement parameters (Å^2^)

**Table:**

	*x*	*y*	*z*	*U*_iso_*/*U*_eq_	
N1	0.18964 (17)	0.6052 (6)	0.1521 (2)	0.0432 (8)	
H1	0.1776	0.6179	0.2126	0.052*	
N2	0.15740 (18)	0.7600 (7)	0.0684 (2)	0.0475 (8)	
O1	0.2584 (2)	0.4124 (5)	0.0501 (2)	0.0577 (8)	
O2	0.20359 (16)	0.4399 (5)	0.3488 (2)	0.0511 (8)	
H2	0.1965	0.4152	0.4084	0.077*	
C1	0.24015 (19)	0.4354 (7)	0.1362 (2)	0.0371 (8)	
C2	0.27283 (19)	0.2675 (7)	0.2264 (3)	0.0369 (8)	
C3	0.25343 (18)	0.2669 (6)	0.3267 (3)	0.0370 (8)	
C4	0.2836 (2)	0.0878 (7)	0.4024 (3)	0.0465 (10)	
H4	0.2699	0.0859	0.4675	0.056*	
C5	0.3333 (2)	−0.0845 (8)	0.3810 (3)	0.0524 (11)	
H5	0.3533	−0.2032	0.4317	0.063*	
C6	0.3545 (2)	−0.0838 (7)	0.2838 (3)	0.0505 (11)	
H6	0.3890	−0.1997	0.2699	0.061*	
C7	0.3240 (2)	0.0891 (7)	0.2088 (3)	0.0422 (9)	
H7	0.3380	0.0871	0.1439	0.051*	
C8	0.1097 (3)	0.9202 (8)	0.0854 (3)	0.0531 (11)	
C9	0.0802 (3)	0.9560 (10)	0.1848 (4)	0.0681 (13)	
H9A	0.0993	0.8241	0.2348	0.082*	
H9B	0.0976	1.1151	0.2170	0.082*	
C10	−0.0029 (3)	0.9511 (13)	0.1616 (5)	0.0863 (18)	
H10A	−0.0200	0.9941	0.2253	0.104*	
H10B	−0.0198	0.7824	0.1415	0.104*	
C11	−0.0380 (3)	1.1323 (12)	0.0730 (5)	0.0874 (17)	
H11A	−0.0917	1.1123	0.0572	0.105*	
H11B	−0.0266	1.3034	0.0961	0.105*	
C12	−0.0081 (3)	1.0816 (13)	−0.0264 (5)	0.0849 (17)	
H12A	−0.0253	0.9184	−0.0543	0.102*	
H12B	−0.0280	1.2059	−0.0795	0.102*	
C13	0.0746 (3)	1.0884 (9)	−0.0060 (4)	0.0690 (14)	
H13A	0.0914	1.2591	0.0096	0.083*	
H13B	0.0908	1.0354	−0.0691	0.083*	

### Atomic displacement parameters (Å^2^)

**Table:**

	*U*^11^	*U*^22^	*U*^33^	*U*^12^	*U*^13^	*U*^23^
N1	0.0527 (19)	0.059 (2)	0.0189 (15)	0.0072 (17)	0.0087 (13)	0.0043 (13)
N2	0.0493 (18)	0.067 (2)	0.0256 (16)	0.0066 (17)	0.0075 (13)	0.0104 (14)
O1	0.082 (2)	0.0684 (18)	0.0267 (14)	0.0165 (16)	0.0210 (13)	0.0059 (12)
O2	0.0656 (18)	0.0681 (18)	0.0219 (13)	0.0188 (15)	0.0142 (12)	0.0037 (12)
C1	0.044 (2)	0.046 (2)	0.0212 (19)	−0.0032 (16)	0.0065 (15)	−0.0014 (14)
C2	0.0394 (19)	0.046 (2)	0.0239 (18)	−0.0062 (16)	0.0031 (14)	−0.0038 (14)
C3	0.0407 (19)	0.0477 (19)	0.0219 (17)	−0.0004 (18)	0.0048 (14)	−0.0020 (15)
C4	0.052 (2)	0.059 (2)	0.029 (2)	0.004 (2)	0.0115 (17)	0.0057 (17)
C5	0.056 (3)	0.056 (2)	0.043 (3)	0.008 (2)	0.0052 (19)	0.0111 (18)
C6	0.052 (2)	0.055 (2)	0.045 (3)	0.0075 (19)	0.014 (2)	−0.0001 (18)
C7	0.048 (2)	0.051 (2)	0.0291 (19)	0.0004 (18)	0.0118 (16)	−0.0045 (16)
C8	0.053 (2)	0.073 (3)	0.033 (2)	0.002 (2)	0.0079 (18)	0.0075 (19)
C9	0.075 (3)	0.086 (3)	0.043 (3)	0.027 (3)	0.012 (2)	0.005 (2)
C10	0.082 (4)	0.110 (5)	0.076 (4)	0.015 (3)	0.037 (3)	−0.001 (3)
C11	0.072 (3)	0.116 (4)	0.072 (4)	0.034 (3)	0.008 (3)	−0.001 (3)
C12	0.075 (4)	0.115 (5)	0.057 (3)	0.023 (3)	−0.002 (3)	0.003 (3)
C13	0.075 (3)	0.076 (3)	0.053 (3)	0.007 (3)	0.007 (2)	0.016 (2)

### Geometric parameters (Å, °)

**Table:**

N1—C1	1.344 (5)	C7—H7	0.9300
N1—N2	1.391 (4)	C8—C9	1.510 (6)
N1—H1	0.8600	C8—C13	1.516 (6)
N2—C8	1.277 (5)	C9—C10	1.493 (8)
O1—C1	1.236 (5)	C9—H9A	0.9700
O2—C3	1.373 (4)	C9—H9B	0.9700
O2—H2	0.8200	C10—C11	1.534 (9)
C1—C2	1.493 (5)	C10—H10A	0.9700
C2—C7	1.391 (5)	C10—H10B	0.9700
C2—C3	1.417 (4)	C11—C12	1.526 (9)
C3—C4	1.398 (5)	C11—H11A	0.9700
C4—C5	1.365 (6)	C11—H11B	0.9700
C4—H4	0.9300	C12—C13	1.486 (8)
C5—C6	1.394 (6)	C12—H12A	0.9700
C5—H5	0.9300	C12—H12B	0.9700
C6—C7	1.370 (6)	C13—H13A	0.9700
C6—H6	0.9300	C13—H13B	0.9700
			
C1—N1—N2	118.4 (3)	C10—C9—H9A	109.4
C1—N1—H1	120.8	C8—C9—H9A	109.4
N2—N1—H1	120.8	C10—C9—H9B	109.4
C8—N2—N1	117.3 (3)	C8—C9—H9B	109.4
C3—O2—H2	109.5	H9A—C9—H9B	108.0
O1—C1—N1	122.2 (3)	C9—C10—C11	112.9 (5)
O1—C1—C2	120.3 (3)	C9—C10—H10A	109.0
N1—C1—C2	117.5 (3)	C11—C10—H10A	109.0
C7—C2—C3	117.3 (3)	C9—C10—H10B	109.0
C7—C2—C1	117.2 (3)	C11—C10—H10B	109.0
C3—C2—C1	125.4 (3)	H10A—C10—H10B	107.8
O2—C3—C4	119.8 (3)	C12—C11—C10	110.4 (5)
O2—C3—C2	119.9 (3)	C12—C11—H11A	109.6
C4—C3—C2	120.3 (3)	C10—C11—H11A	109.6
C5—C4—C3	120.1 (4)	C12—C11—H11B	109.6
C5—C4—H4	119.9	C10—C11—H11B	109.6
C3—C4—H4	119.9	H11A—C11—H11B	108.1
C4—C5—C6	120.5 (4)	C13—C12—C11	112.5 (5)
C4—C5—H5	119.7	C13—C12—H12A	109.1
C6—C5—H5	119.7	C11—C12—H12A	109.1
C7—C6—C5	119.4 (4)	C13—C12—H12B	109.1
C7—C6—H6	120.3	C11—C12—H12B	109.1
C5—C6—H6	120.3	H12A—C12—H12B	107.8
C6—C7—C2	122.3 (4)	C12—C13—C8	112.0 (5)
C6—C7—H7	118.8	C12—C13—H13A	109.2
C2—C7—H7	118.8	C8—C13—H13A	109.2
N2—C8—C9	128.0 (4)	C12—C13—H13B	109.2
N2—C8—C13	117.1 (4)	C8—C13—H13B	109.2
C9—C8—C13	114.8 (4)	H13A—C13—H13B	107.9
C10—C9—C8	111.3 (4)		

### Hydrogen-bond geometry (Å, °)

**Table:**

*D*—H···*A*	*D*—H	H···*A*	*D*···*A*	*D*—H···*A*
N1—H1···O2	0.86	1.97	2.655 (4)	136
O2—H2···O1^i^	0.82	2.15	2.704 (4)	125

Symmetry codes: (i) *x*, −*y*+1, *z*+1/2.
